# Congenital Anterior Urethral Diverticulum in Children: A Case Report and Review

**DOI:** 10.5402/2011/120307

**Published:** 2011-06-14

**Authors:** Y. S. Kadian, K. N. Rattan, Mahavir Singh, Pradeep Kajal

**Affiliations:** Department of Pediatric Surgery, Pt. B.D. Sharma PGIMS, 6/9J, Medical Campus, Rohtak, Haryana 124001, India

## Abstract

Congenital anterior urethral diverticulum (CAUD) is an uncommon condition in children. We describe 2 patients of CAUD who presented with ventral penile swelling; in one, the site of swelling was just proximal to corona which is quite rare. The diagnosis was made on USG and MCU. Both patients had normal renal function. Open diverticulectomy and primary repair was done in both patients.

## 1. Introduction

 Congenital anterior urethral diverticulum (CAUD) is an uncommon condition in children. CAUD is classified into saccular variety and globular variety, the former being more common than the latter [1]. The cause and effect relationship between anterior urethral diverticulum (AUD) and anterior urethral valve (AUV) has been extensively debated. Some authors do not distinguish between AUD and AUV, while others believe these are different entity. The diverticulum that is associated with AUV is not a true diverticulum because in AUD an acute angle is formed between the proximal part of dilated portion and the ventral floor whereas this acute angle is not present in AUV [2]. The diverticulum and valve causes obstruction of male urethra and patient complaints of dysuria, dribbling of urine, recurrent urinary tract infection, or a fluctuant ventral penile swelling [3]. The treatment options in AUD include endoscopic division of distal lip of diverticulum [4], excision of diverticulum with primary repair [5], marsupialisation with staged urethroplasty, or even suprapubic diversion followed by definitive repair [6]. In this paper, we are presenting our experience of two cases of AUD who were managed by excision of diverticulum with primary repair. Moreover, out of these two cases, in one patient, the site of diverticulum was unique because it was just proximal to corona which is a very rare site for its development.

## 2. Case Summaries

### 2.1. Case  1

**Figure 1:**
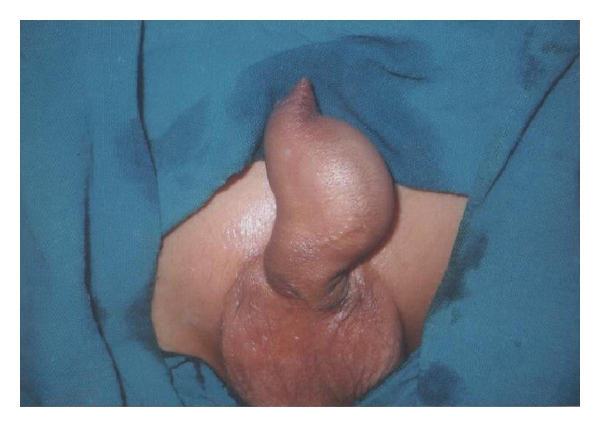
Clinical photograph of the first patient showing a large diverticulum in the anterior urethra.

**Figure 2:**
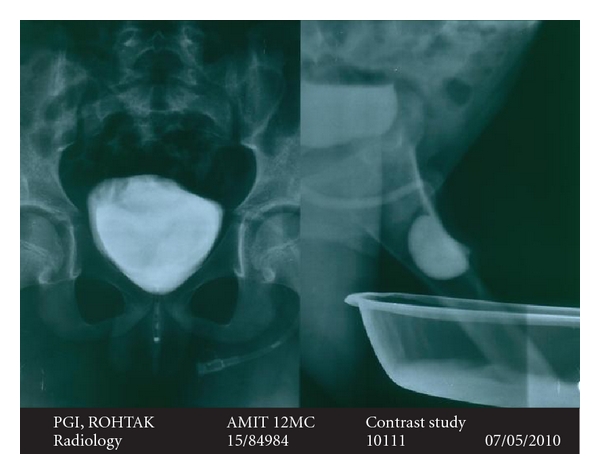
Micturating cystourethrogram showing a diverticulum in the anterior urethra depicting its proximal and distal lip as well as normally filled urinary bladder.

A 12-year-old male presented with complaints of poor urinary stream and a swelling on the ventral aspect of penile urethra (Figure 1). Swelling was a soft cystic, fluctuant, and compressible, and it collapsed completely on manual pressure with urine coming out per urethra. Urine analysis, routine blood counts and blood urea, and serum creatinine were normal. Ultrasonography showed both kidneys normal in size and shape and with normal urinary bladder. Micturating urethrogram was done, and it showed the presence of a wide-mouthed diverticulum in distal penile urethra and the proximal lip of diverticulum forming an acute angle with normal caliber proximal urethra and normal urinary bladder without any vesicoureteric reflux (Figure 2). Patient was managed by open procedure. The diverticulum was opened by incision on the ventral aspect of penile shaft, and its distal lip was widened by abutting a triangular flap taken from one half of the diverticular wall, and the double breasting of urethral suture line was also done using the other half of diverticular wall after scraping its mucosa. Postoperative recovery was uneventful.

### 2.2. Case  2

**Figure 3:**
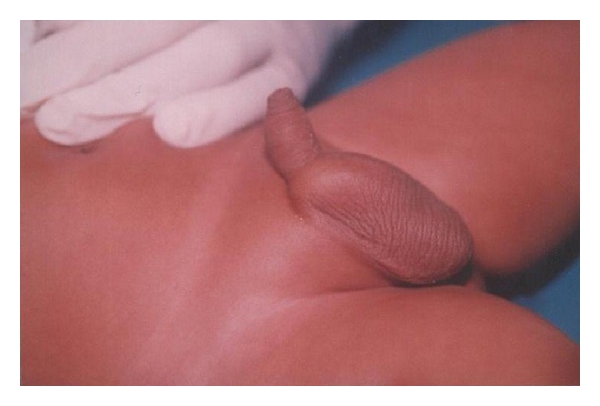
Clinical photograph of the second patient showing a diverticulum at the penoscrotal junction.

A six-month-male child presented with poor urinary stream and a cystic swelling at the penoscrotal junction (Figure 3). On investigations, USG abdomen revealed normal both kidneys as well as urinary bladder. Micturating urethrogram showed anterior urethral diverticulum in midpenile urethra. The patient was managed by open diverticulectomy along with the double breasting of urethral suture line. In the postoperative period, the patient had normal urination without any swelling in the penile urethra and is on regular followup.

## 3. Discussion

Congenital anterior urethral diverticulum (CAUD) may be found all along the anterior urethra but is usually located between the bulbous and the midpenile part. It is rare for AUD to be in distal urethra near the coronal level as in the present case1 of this paper. The embryology of AUD remains unclear. Various proposed hypotheses include a development defect of corpus spongiosum, cystic dilatation of the urethral glands, and sequestration of an epithelial nest after closure of the urethral folds. With a lack of a corpus spongiosum, a urethral dilatation in this region may develop into a diverticulum [7]. Suter proposed the theory that a diverticulum of the urethra develops because of epidermal pockets communicating with the ventral urethral wall. As the anterior urethral tube forms, the urethral groove may leave behind epithelial cells that form a congenital cyst. Cysts in this region developing a communication with the urethra could lead to diverticulum formation as a result of the spontaneous rupture of the cyst into the urethral lumen [8]. 

 AUD may present at any age, from moment of birth upto adult life. Most children with this condition present with difficulty in micturition, dribbling of urine, poor urinary stream, or urinary tract infection. A careful history will reveal that the child never had a good urinary stream since birth, and a tell-tale sign is a cystic swelling at the penile urethra [9]. If it is uninfected and without complication, the mass is unattached to the overlying skin, nontender, and mobile laterally. On compression, urine will be seen dribbling out of the external meatus, and the swelling is seen to empty.

The diagnosis of AUD is usually made by MCUG or retrograde urethrogram. MCUG has the additional advantage of demonstrating proximal changes like megacystis, VUR, or other associated anomaly. VUR has been reported in 20% of patients with AUD [10]. Ultrasonography (USG) is complimentary to the contrast studies to diagnose the condition and offers the additional advantage of evaluating the upper tracts as well. Moreover, voiding USG has been found to be alternative to the contrast studies in making a diagnosis of AUD [11]. Cystourethroscopy is diagnostic as well as therapeutic. A diverticulum typically appears as outpouching from the ventral wall of the urethra and has a proximal and distal rim [4].

 The primary differential diagnostic conditions of AUD include anterior urethral valve (AUV), dilated Cowper's gland ducts, and posttraumatic diverticulum. The presence of a penile or penoscrotal mass clinically and the proximal lip radiologically which is seen as an arcuate filling defect should readily distinguish the diverticulum from the valve. In addition, the proximal lip forms an acute angle with the normal caliber proximal urethra in AUD, whilst in AUV, it forms an obtuse angle [6]. In dilated Cowper's gland ducts, a tubular channel is seen in the ventral surface of the bulbar urethra which it parallels, and its termination is in the urogenital diaphragm [9].

 Treatment of AUD depends on the size of the diverticulum and the degree of obstruction. Transurethral resection (TUR) with a paediatric resectoscope is the treatment of choice for small, well-supported diverticula wherein the distal obstructing lip is resected [4]. Moreover, successful treatment of AUD has also been reported by using a Sachse knife [12]. But in the large diverticula, as also in our cases, open diverticulectomy and primary repair is recommended. We have used the technique of making a triangular flap which is fitted into the distal lip and double breasting of the urethral suture line, as described in literature [5]. Some authors have also advocated the placation of redundant diverticular wall with good results [13]. In situations where there are back-pressure changes of upper tracts with deranged renal function, urinary diversion either by marsupialisation of diverticulum [6] or even suprapubic cystostomy/vesicostomy [14] is a safer option. However, the prognosis depends on the status of the upper tracts.

 To summarise, in patients of AUD with large diverticula without any back-pressure changes, as in the present paper, open diverticulectomy with primary repair is recommended as this procedure carries good results, and it takes care of the redundant diverticular wall.

## Abbreviations

AUD: Anterior urethral diverticulum
AUV: Anterior urethral valves
MCU: Micturating cystourethrogram.
